# Prescription Department

**Published:** 1894-08

**Authors:** 


FOR HEMORRHAGIC DIARRHEA.

℞ Pulverized alum,
Catechu, of each 5 grains.
M and make four pills.
Sig. Three to six pills per day.

℞ Alum,
Catechu, of each 2 grains.
Crude opium, ¼ grain.
Syrup of red roses, q. s.
M. and make one pill.
Sig. Twelve pills per day.

FOR INHALATION.

In bronchitis and tuberculosis Dr. O. B. Douglas, of New York, finds the following mixture very beneficial:

℞ Thymol. gr. x.

Eucalyptol, gtt. xx.

Menthol, gr. xxx.

Ol. cubebs, gtt. xl.

Ol. benzoinol, ℥ iv.

Ol. rose, q. s.

If the mixture be too pungent, it may be reduced by adding plain benoinol.

ANTI-RACHITIC POWDER.

The following mixture is prescribed in Berlin:

℞ Calcii carb. precip., ℥ j.
Calcii phosphat., ℥ ss.
Ferri lactat., gr. xlv.
Sacch. lact., ℥ iss.
M. Sig. Dose according to age.—*La Médecine Moderne. Ex.*

FOR CHRONIC CATARRHAL DIARRHEA.

℞ Extract of rhatany, 1 ʒ.

Sryup of morphine,

Syrup of quince, each 12 ʒ.

Lime water, q. s. to 5 ℥.

℞ Liq. perchlor. of iron, 8–15 grs.
Sydenham's laudanum, 20 to 30 gtt.
Mucilage of gum arabic,
Simple syrup, of each 1 ℥.
Cinnamon distilled water, q. s. to 5 ℥.

Sig. Dessertspoonful every two hours of either of the last two mixtures.—*Therapeutic Gazette.*

DIPHTHERIA.

Professor J. C. Wilson recommends as a spray:

℞ Olei eucalypti, f ʒ ij.

Sodii benzoatis, ʒ j.

Sodii bicarbonatis ʒ ij.

Glycerini, f ℥ ij.

Aquæ calcis, q. s. ad. O ij.

Sig. Apply as a spray to the membranes every half hour from three to five minutes at a time.--*Medical World.*

PAINFUL DYSPEPSIA.

℞ Bismuthi subnitrat., gr. x.

Magnes. carbonat., gr. xv.

Liq. potassæ, ♏ x.

Acid. hydrocyan. dil., ♏ iij.

Tinct. zingiberis, ♏ v.

Aq. menth. pip., q.s. ad. f ℥ j—M.

For one dose. To be repeated two or three times daily. Shake well.—*Therapeutic Gazette.*

HEADACHE.

℞ Caffeinæ citratis, gr. x.

Acetanilidi, gr. xxx.

Sodii bromidi, ʒ j.

Sodii bicarbonatis, ℈ ij.

Div. in chart. x.

Sig. One powder, repeated if necessary, one hour.

TÆNIA.

Dr. Descroizilles prescribes the following mixture:

℞ Ethereal extract of male fern, ʒ ij.

Calomel, gr. vj.

Peppermint water, ʒ iiss.

Gum arabic, ʒ i½.

Simple syrup, ʒ v.

Distilled water, q. s. ad. ℥ ij.—M.

—*La Médecine Moderne. Ex.*

FOR NEURALGIA.

A pill, made by the following formula, given three times a day is recommended in cases of neuralgia:

℞ Iron tartrate, gr. ij.
Quinine sulphate, gr. ij.
Tartaric acid, gr. ss.
Extract of nux vomica, gr. ss.

—*Druggists' Circular.*

